# IOCBIO Kinetics: An open-source software solution for analysis of data traces

**DOI:** 10.1371/journal.pcbi.1008475

**Published:** 2020-12-22

**Authors:** Marko Vendelin, Martin Laasmaa, Mari Kalda, Jelena Branovets, Niina Karro, Karina Barsunova, Rikke Birkedal

**Affiliations:** Laboratory of Systems Biology, Department of Cybernetics, School of Science, Tallinn University of Technology, Tallinn, Estonia; bioinformatics, GERMANY

## Abstract

Biological measurements frequently involve measuring parameters as a function of time, space, or frequency. Later, during the analysis phase of the study, the researcher splits the recorded data trace into smaller sections, analyzes each section separately by finding a mean or fitting against a specified function, and uses the analysis results in the study. Here, we present the software that allows to analyze these data traces in a manner that ensures repeatability of the analysis and simplifies the application of FAIR (findability, accessibility, interoperability, and reusability) principles in such studies. At the same time, it simplifies the routine data analysis pipeline and gives access to a fast overview of the analysis results. For that, the software supports reading the raw data, processing the data as specified in the protocol, and storing all intermediate results in the laboratory database. The software can be extended by study- or hardware-specific modules to provide the required data import and analysis facilities. To simplify the development of the data entry web interfaces, that can be used to enter data describing the experiments, we released a web framework with an example implementation of such a site. The software is covered by open-source license and is available through several online channels.

## Author summary

In biological and other types of experiments, we frequently record changes of some parameters in time or space. It is common to analyze the data by splitting the recording into smaller sections and relating it to some changes induced by the researchers. The steps involved in the analysis are: splitting of the data, fitting them to some function, relating the fit result to the change in the environment, and normalization. These steps are frequently done through several software packages, spreedsheets, and manual copy and paste between the programs. The software presented in this work allows to make all these analysis steps in one database in a manner that is easy, can be reproduced by others, and clearly tracks the history of all the analysis steps. In addition, it allows to link the experimental data with the description of the experiment, making it simple to perform tasks such as normalization of the recorded values, relating experimental recordings to the sample or animal, as well as extracting data from the laboratory database for publishing. The software is written to be easily extendable by user-defined modules to fit the analysis pipelines and is expected to improve the data analysis practices in research.

This is a *PLOS Computational Biology* Software paper.

## Introduction

The first step towards the application of FAIR (findability, accessibility, interoperability, and reusability) principles [1], is to ensure that the scientific records are organized and stored in a manner that would allow to later find and reuse the original data as well as trace back the steps used to analyze them. In addition to FAIR principles, it is a vital requirement to ensure repeatability of studies in response to the replication issues raised in many fields [2]. While several specialized repositories exist ensuring storage and reusability of the data stored within them, a large fraction of traditional bench science datasets do not fit such specialized repositories [1]. Hence, there is a need for heterogeneous data repositories and an ability to record datasets together with the metadata, i. e. data describing these datasets. Several open source laboratory information management systems (LIMS) have been developed to keep the track of experimental settings and relate them to the measurements [3–8]. However, due to the large diversity of the experiments possible to track by LIMS, the measurements are frequently stored as raw data together with the analysis of it, but the intermediate steps applied by researcher are separate. The software described in this work is aimed at complementing LIMS and provides the mechanisms of the primary analysis (defined below) of the data in reproducible form. In addition, software is provided to simplify the transition from spreadsheet-based data analysis pipeline to the database-based pipeline for the laboratories that require this transition.

In the fields that we work, we are commonly recording changes in some parameters in time or space. Later, the original data traces are fitted with some simple functions or averaged in different time intervals describing the response of the cells to treatment or external stimuli. For example, from the recording of the changes in oxygen concentration in solution, we determine respiration rate (derivative of the oxygen concentration change in time) under different conditions to study different aspects of cellular bioenergetics [9]. For respiration rate analysis, the data analysis pipeline frequently involves selection of regions of interest which are fitted to determine respiration rate (primary analysis) and fitting the obtained respiration rate changes as a function of metabolite concentration (secondary analysis). Later, the obtained respiration rates are frequently normalized by protein content or marker enzyme activity using metadata, such as volume of cell suspension used in the measurements, linked to the experiment. While the software was first applied for respiration rate measurements, we found out that a large fraction of our experiments require a similar pipeline of analysis: calcium-induced fluorescence oscillations, activity of ion channels under different conditions, absorbance changes induced by enzymatic systems, changes in sarcomere length during cardiac contractions. So, what was originally planned as a specific tool for analysis of respiration measurements, was adopted to most of the analysis pipelines in the laboratory and is now released for general use.

The aim of the presented software is to analyze data traces according to the described protocols, store the intermediate results in the database and allow to link these results to the experimental metadata. The implemented software consists of a modular Python program IOCBIO Kinetics that runs on different desktop platforms and a web framework IOCBIO WebDB, for laboratories not using LIMS, to simplify the creation of metadata entry web sites that can be used next to the bench. This software aims to streamline data entry, analysis, and storage of the data within the laboratory and has to be complemented with the other software for the next step in application of FAIR principles, which is to transform the data into standard-based machine-findable form and publishing it in the public databases.

## Design and implementation

### IOCBIO Kinetics

The application is separated into the core and the modules. The core is handling the graphical user interface (GUI), communication with the database, communication between modules and GUI, and providing base classes for data analysis. The modules are loaded during startup of the application by the core and are used to import experimental data, analyze primary experimental data and, if needed, analyze results obtained from the analysis of primary experimental data. Communication between primary and secondary analysis occurs through the database backend preserving intermediate analysis results.

The application core is responsible for loading the modules which are included in the main source code of the application as well as the user provided folders. Module loading is written in a way that allows users to simply extend the functionality of the application to cover their specific experiments.

Several modules used for data analysis are provided: mean, median, and standard deviation for region of interest, linear regression, fitting against Michaelis-Menten relationship, and a few others. The library of these helper modules can be extended further as required.

In addition to the application, scripts assisting data fetching from the database are provided. These scripts take an SQL query and return the resulting table in long or wide formats or perform statistical analysis using Bayes ANOVA through BayesFactor package [10].

The use of the program requires a database configured to allow access of the user. The data analysis modules will create missing tables and views if needed.

The application is written in PyQt ensuring support for all main desktop operating systems (used on Linux and Microsoft Windows, tested on MacOS). Communications with the database is realized through Python package Records together with SQLAlchemy. Graphs are plotted using PyQtGraph with the analysis supported by SciPy and NumPy. All the used libraries are given in the included setup script.

### IOCBIO WebDB

The released web framework allows setting up a simple web site for entering metadata. Its functionality is set by the administrator through a routing table, the set of Python classes, and SQL commands for fetching data. The classes describing the site are simple and each contain a description of a data table and its relation to the other tables. The site used by our laboratory is provided as an example to simplify setting up a new one. Specific requirements and installation instructions are given in the documentation.

IOCBIO WebDB is based on Pyramid web framework and communicates with the database through through Python package Records together with SQLAlchemy.

## Results

### User interface

**Fig 1:**
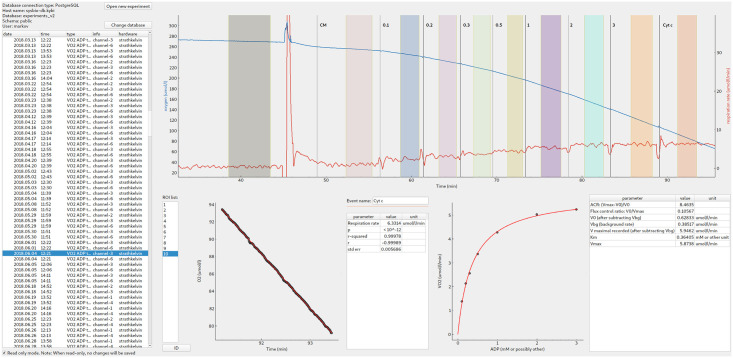
Overview of the graphical user interface. See main text for description.

As shown in Fig 1, the user interface shows a list of experiments available for analysis (left) and the views of the selected experiment (right). On the top part of the current experiment view, there is a full experimental trace which the user can scroll and scale. In this part, the user can create new regions of interest (shown in color), move and change them. If applicable, the data in the selected region of interest will be fitted with the fit result shown on the bottom left graph. The resulting fit parameters will be shown in the table next to it. These are the results of the primary data analysis.

In such experiments where the response of reaction rate to the changes in concentration is studied, it is possible to process the results of the primary analysis by secondary analysis modules. In the example shown in Fig 1, the respiration rate changes induced by increase of ADP in the solution surrounding permeabilized cardiomyocytes [11] are fitted with the Michaelis-Menten relationship, graph on the right bottom. The obtained dissociation constant and maximal rate are shown in the table next to the graph together with the other secondary analysis results, such as flux control ratio in the experiment.

Behind the scenes, all the analysis data are stored in the database together with the information sufficient to show the original data trace. When browsing the data entered earlier by switching the datasets using the list on the left of Fig 1, the application works in read-only mode by default. This can be changed using a toggle on the bottom left (Fig 1).

### Database structure

The application core generates two main tables (experiment and roi) and table iocbio for database schema updates. Experiment and region of interest (ROI) tables define records for each experiment (one per experiment) and each ROI linked to the experiment via a reference. The rest of the tables and views are generated by the modules and, depending on implementation, include results linked either to a specific ROI (for example, mean value) or a specific experiment (for example the ratio of rates obtained under different conditions).

### Modules

While the application core provides facilities to make the analysis possible, the data are analyzed by the modules. The modules are operating within the defined application programming interface and are called by the application according to their type. Modules are of four different types in accordance with their task: defining program arguments, creating required database tables and views, data import, and data analysis. Below, we will focus on the main modules covering data import and analysis.

The data import module should be able to create an internal Data object describing the dataset either through a given experiment ID or from the raw data. It is recommended, if feasible, to let this module store the imported raw data in one of the database tables. When importing the raw data from the file produced by the measurement device, the module has to generate as experiment ID. The generated experiment ID has to be the same for the same dataset to avoid multiple imports. In practice, we use some of the hashes to produce experiment IDs.

The intermediate object of type Data is passed between the modules and contains one or more traces. The traces are expected to have the same type of argument (time or space, not mixed), but can be recorded at different argument values (for example, recordings of several variables at different time-moments). The program supports splitting of the Data object into regions of interest. The split can be the same for all traces or made separately, as defined by the analysis modules. An example, where separate regions of interest are required, is in studies of cardiac contractility. In this case, sarcomere shortening recorded by a fast transmission camera can be analyzed in time-moments which are different from the time-moments with calcium induced fluorescence changes, recorded by another camera.

The analysis modules receive the Data object and the primary data analysis modules are expected to handle events associated with the dataset if there are any. For example, during respiration experiments, the researcher may enter comments to mark events. The primary analysis modules can assist with automatic split of the trace on the basis of the events or some other protocol specification. In particular, it could be important for the experiments that can have hundreds of regions of interest, such as some electrophysiological recordings [12].

The primary and the secondary analysis modules record their analysis results into the database and, in the case of the secondary analysis modules, are expected to use the results of the primary analysis from the database. The analysis modules are expected to be derived from the fitting modules provided by the application core classes. This frequently allows to write analysis modules with a minimal amount of code by delegating most of the operations to the parent class and mainly defining which database tables to use.

Together with the program, we distribute the modules used in our laboratory as examples that can be used to derive new modules.

## Availability and future directions

The presented software allows to analyze data traces in a modular way and store the data analysis results in a database. With the changes in analysis pipeline, as discussed below, it will simplify the application of FAIR principles [1] in the laboratories using the software and, through recording of all steps, ensure that the analysis part of the study is reproducible. At the same time, it targets simplification of the routine workflow by the researchers when analyzing the data.

The written software is expected to be used together with further analysis steps through the database. While many laboratories do use databases for their data analysis, it is not as common as it should be. While writing the application, care was taken to simplify the entry for laboratories that are routinely using spreadsheets in their data analysis.

Below, we discuss the recommended analysis pipeline, required investment to use the software, compare with the other approaches and outline the future directions.

### Availability

IOCBIO Kinetics is distributed through multiple channels. The manuals and links are available at https://iocbio.gitlab.io/kinetics with the application available through PyPi and Anaconda. IOCBIO WebDB repository is accessible at https://gitlab.com/iocbio/webdb.

### Analysis pipeline

We recommend to organize the data analysis pipeline reflecting all the steps done by the researchers from the raw data to the processed values as closely as possible. In addition to the data fitting, as covered by Kinetics software, it frequently involves normalization to protein content, sample mass, or similar. These normalization steps can be made using the facilities provided by the databases leading to the view of the data in normalized form that can be analyzed further using statistical analysis software.

For those who have not worked with databases before, the relational databases, as the one used in this work, keep the data in the tables. Data between tables can be linked, usually through IDs associated with each record. The processing of the data and presentation of the data in different form can be done through views. So, in spreadsheet analogy, views will be the representation of the data which had formulas applied.

**Fig 2:**
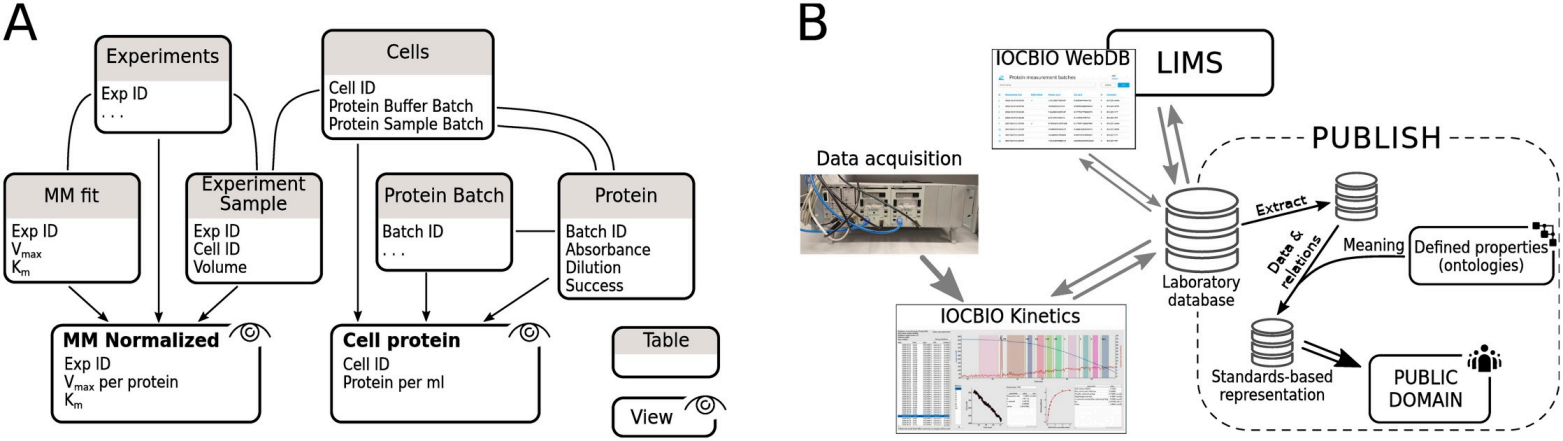
Example analysis pipeline (A) and overall positioning of the software in experimental data processing and publishing (B). In A, database tables are shown as boxes and relations between the tables as lines. Views are formed as shown by the arrows. See main text for description of the example pipeline. In B, the positioning of the released software relative to the laboratory data handling together with the publishing of the data is shown.

Let us describe the recommended analysis pipeline by an example in respiration analysis where we want to record respiration rate changes in response to changes in ADP in the solution surrounding permeabilized cardiomyocytes. An example is illustrated in Fig 2A and explained in details below. Kinetics software analysis would lead to the tables with respiration rate under different conditions, the apparent maximal respiration and apparent affinity coefficient as a result of the Michaelis-Menten fit, and the table with maximal and minimal respiration rates. Here, respiration rates are normalized by chamber volume as this comes with the hardware that produced the raw traces, but not to the sample-oriented quantity.

To normalize respiration rate to the protein content, we need additional data. First, we need a table linking experiment ID, as generated by Kinetics software, with the sample ID and the sample volume used in that particular experiment, as provided by the researcher. Next, we need the protein content associated with the sample ID. As protein content is usually measured in batches (one batch for sample and another for buffer), we process protein measurements through the database as well. Namely, we insert each protein sample recording as a part of a batch and tag whether the sample is considered successful. As a result, calculation of the mean protein content in the sample and the buffer is done by the database view. Now, to get the protein content associated with the sample ID, we just need to associate sample ID with the protein measurement batches (one sample and one buffer). For the entered data, protein content for sample ID will be calculated automatically and can be used for normalization in the database view.

In our case, to get the normalized respiration rate, the rate in the raw data table will have to be multiplied by the respiration chamber volume, divided by the amount of sample volume and by the protein content per sample volume. The values which do not require any additional normalization (apparent affinity of the respiration and flux control coefficient) can be carried over directly to the summarizing view describing the experiment making it simple to access all the required data through the same database view. The presented example is illustrated in Fig 2A.

### Comparison with spreadsheet-based analysis

When compared to spreadsheet-based analysis, the analysis pipeline has similar or the same steps. The major difference is in the absence of manual copy-and-paste operation that is prevalent when data are analyzed by spreadsheets. As a result, the risk of mistakes done routinely is minimized to the data entry. As more data are analyzed using the same views in the database, finding mistakes in the formulation of the views is easier as well. When errors are found, they can be fixed centrally and without manual recheck of the formulas in all spreadsheets involved in the study. So, in addition to advantages given by the central storage of the data, the analysis using databases is expected to be less error-prone and to lead to a better quality of the data in the study. Moreover, many researchers (from the same or different labs) can easily view the same raw data and also contribute to the same experimental data collection and analysis while being sure that the data are analyzed the same way.

### Required investment

To use the software, specialized data analysis modules have to be written. To simplify it, we wrote a detailed documentation describing how to make the modules. Over time, we expect that the library of modules will be extended by the modules written together with hardware manufacturers and interested researchers. On our side, we will provide as much assistance as we can in writing the required modules.

For the laboratories not used to analyze data through a database, there are few additional investments involved. The main investment is learning how to access the database and how to use Structured Query Language (SQL) to query the database and write views. From experience, a new user, who does not have any particular background in programming, can learn SQL required to fetch and analyze the data in 2-3 days. Obviously, it takes longer for more advanced use, but, with the large amount of supporting information available online, the skills can be acquired while solving practical problems.

In addition to the education of the researchers, the database has to be set up. It is also recommended to set up the web interface to access and enter the data. The procedures for such a setup are standard and the local IT support is expected to be able to perform it. For the metadata entry, either LIMS or the released IOCBIO WebDB can be used, depending on the preference of the local IT support.

### Positioning of the software

The released software covers a specific part in the overall scheme of data analysis and publishing (Fig 2B). It is focused on simplification, reproducibility, and, as explained below, assists with the implementation of FAIR principles in the data analysis pipeline for experiments with data traces. The software targets the data handling within a research team. As the experiment and ROI IDs are unique, they are ready for deployment of the relevant datasets into global databases covering similar studies run by several teams as well. These IDs are also a means to linking the data analyzed by the software with the other data in the database including the metadata describing the experiment. As explained in the example analysis pipeline above, the experiment ID is expected to be linked to the sample used in the experiment and its protocol description.

In part, IOCBIO WebDB is overlapping with the more comprehensive LIMS software. As it is mainly developed for simple data entry, it maybe easier to use next to the bench than the full LIMS stack. In addition, it can simplify the transition to the database-based data analysis pipeline, a requirement that we expect too many laboratories still have.

The released software is a part of a software infrastructure required for ensuring applicability of FAIR principles. To make data from the laboratory database public and available according to FAIR principles, several additional steps have to be performed. As the laboratory database is expected to have records not related to the published study, one has to extract the related data. As described in the User Manual, we recommend to use tools which allow to extract a subset of database data according to the user specified model while keeping relations between data intact. Combined with Kinetics software version information and source code for all the external modules developed for this analysis, such extracted data together with experimental files not incorporated into the database will form a full dataset that can be reanalyzed and the analysis steps reproduced.

With the data extracted, the next step is to make it FAIR. In principle, it requires description of the data, i.e. adding meaning for data values and relationships between records in the different database tables, using standards. Such a description of the data is not a part of our software package as we rely on other tools for it. For data description, several standards exists and, as a minimum, data have to be described during the upload to the repositories. However, as data form a database, it is possible to convert it to a Resource Description Framework (RDF) and associated Semantic Web technology stack, used by linkedISA [13]. The tools for conversion of rational databases into RDF are developed as a part of Semantic Web initiatives [14]. Combined with the layers defining the meaning of relationships and data, data can be made machine findable [1, 13]. We plan to look into how to simplify the process by adding information regarding the meaning of the data and the relationship between records in the database generated by our software. We expect to add that as a part of the modules API, but the task is not trivial as it would require using the same approach as the rest of the software stack, such as LIMS, used by the laboratories.

The developed software has prospect as a tool to make thorough comparisons between laboratories, which, in turn, will reduce the need for replication and the number of animal experiments. The software is expected to facilitate collaboration between researchers within the same and different laboratories during active phase of the study and simplify preparation of the raw data for big data analysis.
